# Investigating the Impact of Humic Acid on Copper Accumulation in *Sinonovacula constricta* Using a Toxicokinetic–Toxicodynamic Model

**DOI:** 10.3390/toxics12010074

**Published:** 2024-01-15

**Authors:** Mingyi Cai, Tian Ma, Huayong Que, Bo Shi, Xiande Liu, Yizhou Ke

**Affiliations:** 1 Key Laboratory of Healthy Mariculture for the East China Sea, Ministry of Agriculture and Rural Affairs, Jimei University, Xiamen 361102, China; myicai@jmu.edu.cn (M.C.); mt9812@139.com (T.M.); hque@jmu.edu.cn (H.Q.); shibo@jmu.edu.cn (B.S.); xdliu@jmu.edu.cn (X.L.); 2 State Key Laboratory of Mariculture Breeding, Fisheries College of Jimei University, Xiamen 361102, China

**Keywords:** copper, humic acid, toxicokinetic–toxicodynamic model, *Sinonovacula constricta*

## Abstract

In aquatic ecosystems, the interaction between heavy metals and dissolved organic carbon (DOC) plays a pivotal role in modifying the bioavailability of these metals. This study, employing a toxicokinetic–toxicodynamic model, delves into the interactive effects of humic acid (HA), a significant component of DOC, on the bioaccumulation and toxicity of copper (Cu) in the estuarine economic bivalve *Sinonovacula constricta*. Utilizing the stable isotope ^65^Cu as a tracer, we evaluated Cu uptake in *S. constricta* under varied DOC concentrations in a controlled laboratory setting. Our findings reveal that at DOC concentrations below 3.05 mg L^−1^, the bioavailability of Cu is reduced due to shifts in the speciation distribution of Cu, resulting in decreased bioaccumulation within *S. constricta*. Conversely, at DOC levels exceeding 3.05 mg L^−1^, the formation of colloidal Cu–HA complexes allows its entry into the bivalves’ digestive system. Moreover, toxicity assays demonstrate an increase in *S. constricta* survival rates with higher DOC concentrations, suggesting a protective effect of DOC against Cu toxicity. The integration of accumulation and toxicity data infers that Cu–HA complexes, when ingested via the digestive tract, exhibit lower toxicity compared to Cu directly assimilated from the water phase. These findings emphasize the need to consider environmental DOC levels in assessing Cu pollution risks and provide insights for managing heavy metal toxicity in estuarine aquaculture.

## 1. Introduction

As a prevalent heavy metal contaminant in China’s coastal waters [1,2], copper (Cu) is readily absorbed and utilized by marine organisms, particularly bivalves, leading to significant accumulation in their tissues [3,4]. This accumulation escalates with increasing Cu levels in the water, particularly in heavy metal-polluted estuarine areas [5]. Furthermore, Cu is more toxic to bivalves than many other heavy metals [6]; when tissue concentrations exceed certain threshold levels, it can result in toxic effects [7,8], thereby posing substantial risks to marine bivalve populations.

It is worth noting that the chemical composition of seawater, such as dissolved organic matter (DOM), can greatly influence the bioavailability of dissolved Cu [9,10]. DOM is typically characterized by the concentration of dissolved organic carbon (DOC). According to the concept of the widely used biotic ligand model (BLM), aquatic organisms have the highest utilization rate for free ion metals in water, while DOC competes with free ion metals for binding, thereby reducing the proportion of free metals in water and consequently decreasing their bioavailability [11,12]. Theoretically, the higher the concentration of DOC in the water, the lower the utilization rate of heavy metals by organisms, and the lower toxicity to organisms. Humic acid (HA) is a significant component of DOC found in natural water bodies [13]. Roughly half of the dissolved organic matter in natural waters consists of HA [14]. Humic substances demonstrate a notably high affinity for Cu particularly due to its natural tendency to form carbonate and hydroxide complexes [15,16,17].

Previous studies have reported on the influence of DOC in water bodies, including HA and other small molecules, on the accumulation of metals in bivalves. The biokinetic (BK) model approach, which is only used with bioaccumulation, has been successfully applied in these studies [18,19,20,21]. However, there are still few studies that connect the effects of Cu–DOC complexes on the bioaccumulation and toxicity of Cu in marine bivalves [22]. The toxicokinetic–toxicodynamic (TK–TD) model offers a robust framework for examining these relationships by simulating the dynamic process of accumulation and its toxic effects in organisms [23]. In this approach, toxicokinetic (TK) models link time-dependent bioaccumulation with the concentration in the exposure medium, encompassing uptake, distribution, metabolism, elimination, and growth dilution [24]. Meanwhile, toxicodynamic (TD) models correlate the accumulated concentration with the progression of toxic effects, such as mortality. 

*Sinonovacula constricta*, a type of clam and one of China’s four traditionally farmed bivalves, is extensively distributed along the nation’s coast and is a key contributor to the marine bivalve aquaculture industry, boasting an annual production of approximately 850,000 tons in 2022 [25]. However, since domestic *S. constricta* aquaculture sites are mainly located in heavily human-impacted nearshore estuaries, there is a higher risk of heavy metal accumulation and subsequent food safety issues in *S. constricta* aquaculture [26]. Overall, studying the accumulation process of Cu in *S. constricta* and its limiting factors in aquaculture waters is of great significance for food safety control and ecological risk assessment in *S. constricta* aquaculture.

In this study, we utilized the stable isotope ^65^Cu as a tracer; we not only examined the effects of varying concentrations of HA on Cu uptake in a controlled lab environment but also conducted toxicity tests. A one-compartment TK–TD model was established to elucidate the role of DOC in influencing Cu bioavailability and its resultant toxicity to *S. constricta*. This study could offer insights for ecological risk assessment and the management of heavy metal pollution in aquaculture settings.

## 2. Materials and Methods

### 2.1. Clam Acquisition and Acclimation

The razor clams *S. constricta* used in the experiment, with their gonads in the undeveloped stage, were collected from an aquaculture farm (23°56′02.1″ N, 117°23′39.7″ E) in Yunxiao, Fujian Province, China, in March 2021. They were temporarily cultured in a laboratory environment for 5–7 days before experiments began. The formula for the artificial seawater was adapted from previous literature [21]. The seawater was prepared by mixing sea salt with Milli-Q water to achieve a salinity of 15. This seawater composition was then utilized for all subsequent experiments. The background value of Cu in the artificial seawater is less than 1 µg L^−1^. During this period, the clams were fed with *Chlorella* sp. microalgae once a day, with a feeding amount of 2–3% of their wet weight. During this period, and throughout the entire experiment, the temperature of the seawater was consistently maintained at 20 ± 1 °C and the dissolved oxygen content was always kept above 6 mg L^−1^.

### 2.2. Cu Accumulation and Elimination

The reconstituted seawater was filtered through 0.22 μm polypropylene membranes (Calyx Capsule) and 400 W UV lamps were used to degrade the background DOC in the filtered seawater for 8 h. The seawater was divided into 4 groups to which were added different nominal concentrations of HA (Sigma Aldrich) (0, 5, 10, 20 mg L^−1^); each group had 3 replicates. Following the addition of HA, the prepared seawater was sequentially filtered again through polypropylene membranes (Calyx Capsule) with pore sizes of 2 μm and subsequently 0.22 μm to effectively remove particulate matter. Prior to initiating the experiment, for each gradient of HA, 30 mL water was sampled in a brown reagent bottle for DOC determination. In this study, the stable isotope ^65^Cu (Trace Science International, Canada) was used as a tracer. The lowest Cu concentration (15 μg L^−1^) used for the experiments was commonly found in estuaries in China [4,5]. The clams were exposed to the isotope ^65^Cu at a nominal concentration of 15 and 150 μg L^−1^ (see Table S1 for measured concentrations). Seawater for exposure, spiked with ^65^Cu, was allowed to equilibrate for one hour prior to commencing the uptake experiments. In preparation for the exposure experiments, the 4 L polypropylene containers were soaked overnight in 5% HNO_3_ and then thoroughly rinsed three times with Milli-Q water.

After the start of the experiment, 22 clams were placed in the 4 L polypropylene containers filled with 4 L of prepared exposure medium and exposed for 12 h in the absence of supplemental feeding. During the initial 12 h exposure period, samples were taken every 3 h, with 3 mL of water and 2 clams for each replicate at each sample time. After the exposure, clams were transferred to clean filtered seawater for a 168 h depuration phase. The 3 mL water samples of each replicate from the same group were combined and acidified with 90 µL of 65% HNO_3_ for subsequent chemical analysis. In the depuration stage, the clams were provided with daily feeding, consistent with the protocol established during the acclimation period. They were then sampled at intervals of 12, 24, 48, 72, 120, and 168 h specifically for the group exposed to 15 μg L^−1^ of ^65^Cu during the uptake phase, with each sampling consisting of 2 clams per replicate. Throughout the entire duration of the experiment, all clam samples were carefully dissected, and soft tissues were rinsed twice with 1 mmol L^−1^ EDTA, followed by two rinses with Milli-Q water to minimize surface adsorption of ^65^Cu. The soft tissues were then placed in individual self-sealing bags, freeze-dried for 48 h, and weighed. The freeze-dried tissues were added to 15 mL centrifuge tubes with 1 mL of HNO_3_ for digestion, which was carried out at room temperature for 8–12 h, followed by hot digestion in a digestion instrument for 24 h at a temperature of 80 ℃. 

### 2.3. Cu Toxicity Test

A short-term toxicity test was conducted under a high ^65^Cu concentration (nominal concentration of 300 µg L^−1^) for 96 h. The 4 L polypropylene containers were pre-treated by soaking overnight in 5% HNO_3_ and subsequently rinsed three times with Milli-Q water before use. The clams were exposed to the gradient DOC seawater prepared as mentioned above (Section 2.2). Each group had 3 replicates, and a group not spiked with any Cu was set as the control group; each replicate contained 4 L of exposure solution and 20 individuals. The accumulation experiments under 300 µg/L ^65^Cu (see Table S1 for measured concentrations) were conducted simultaneously and shared the same container with the toxic test. In the first 12 h of the toxicity test, sampling occurred every 3 h, with each sample comprising 3 mL of water and 2 clams. The samples were processed as described in Section 2.2. Throughout the duration of the toxicity test, the exposure medium was not replaced. The experiment results were considered reliable only when the survival rate of the control group was above 90%. Throughout the experiment, the mortality of the clams was recorded at intervals of 6–8 h, and deceased specimens were promptly removed from the containers. A clam was considered deceased if its shell remained open and it was unresponsive to touch.

### 2.4. Chemical Analysis

The Cu concentrations in water and organisms were determined using Inductively Coupled Plasma Mass Spectrometry (ICP-MS, NexION 2000). A calibration standard (Agilent 5188-6525) was used for calibration and quality control by measuring a control standard for every 10 samples. An internal standard Ge was used during the measurement, and it was checked every 10 samples to ensure the stability of the internal standard between 90% and 110%. The obtained results were corrected using the internal standard. The standard reference material (SRM 1566b, oyster tissue) was digested and analyzed, and the results showed that the Cu recovery rate was within the range of 90–110%.

For the analysis of DOC, water samples were treated with H_3_PO_4_ to lower their pH to below 2 and then preserved in amber glass bottles at 4 °C. DOC concentrations were quantified using a Shimadzu TOC-Vcph total organic carbon analyzer.

### 2.5. Toxicokinetic–Toxicodynamic Modeling and Parameters Estimation

#### 2.5.1. Toxicokinetics

To simulate the ^65^Cu accumulation and elimination process in *S. constricta*, a one-compartment toxicokinetic model was established, drawing on methodologies established in previous studies [7,8]:

(1) $$\frac{{dC}_{int}\left(t\right)}{dt}=k_{u}\times C_{w}\left(t\right)-k_{e}\times C_{int}\left(t\right)$$

$C_{int}\left(t\right)$ represents the accumulated concentration of ^65^Cu in clams at time t (µg g^−1^); $C_{w}$ (µg L^−1^) is the Cu concentration in the water; $k_{u}$ is the uptake rate constant (L g^−1^ d^−1^); and $k_{e}$ is the elimination rate constant (d^−1^). $J_{int}\left(t\right)$ is the uptake rate of the clams and $J_{int}\left(t\right)$ changes with the variation of Cu concentration ( $C_{w})$ in the water. $J_{int}\left(t\right)$ and $C_{w}$ have the following relationship:

(2) $$J_{int}\left(t\right)=k_{u}\times C_{w}\left(t\right)$$

#### 2.5.2. Toxicodynamics

The survivorship of clams in the toxicity experiments was characterized through the application of the toxicodynamic model, drawing on methodologies established in previous studies [7,8]:

(3) $$\frac{dH\left(t\right)}{dt}=\left\{\begin{array}{c} k_{k}\times {(C}_{int}\left(t\right)-C_{IT})+h_{0}\;if\;C_{int}\left(t\right)>C_{IT}\\ h_{0}\;otherwise\; \end{array}\right.\;$$

(4) $$S^{\left(t\right)}=e^{-H(t)}\;$$

(5) $$S_{0}(t)=e^{{-h}_{o}\times t}\;$$

$H\left(t\right)$ represents the hazard posed by Cu exposure; the mortality rate, denoted by $k_{k}\;$ (g µg^−1^ h^−1^), quantifies the mass of organisms that perished per microgram of Cu per hour; $C_{IT}$ is the threshold concentration that causes toxicity; $h_{0}$ is the background hazard rate (h^−1^), which is assumed to be zero in this study due to the absence of mortality in the control group. $S\left(t\right)$ is the survival probability of the organisms at a given time t; $S_{0}\left(t\right)$ is the survival probability of the organisms in the negative control. The core concept of this model is that the hazard of Cu exposure begins to accumulate once the Cu concentration in the organism’s tissues surpasses the threshold ( $C_{IT}$ ). The hazard rate is directly proportional to the excessive accumulation of Cu above the threshold, or [ $C_{int}\left(t\right)$ − $C_{IT}$ ].

#### 2.5.3. Parameters Estimation

The TK–TD model is established and relevant parameters are calculated using the Openmodel 2.4.3 software (Neil Crout at Nottingham University). To calculate the TK model parameters, the uptake and elimination data are fitted using Equations (1) and (2); to calculate the TD model parameters, Equations (3) and (4) are used for fitting. The parameters and standard deviations for TK and TD are calculated using the Marquardt algorithm.

### 2.6. Data Analysis

For the accumulation experiment, samples were analyzed for both ^63^Cu and ^65^Cu, and the actual ^65^Cu concentration was obtained by subtracting the background value [27]:

[65Cunew]=([65Cu]− [63Cu]) · F65

The term [^65^Cu] represents the aggregate concentration of isotope ^65^Cu within the samples, while [^63^Cu] denotes the background level of isotope ^65^Cu. Here, ^65^F refers to the natural isotopic abundance of ^65^Cu, which is valued at 0.3085.

To evaluate variations in Cu concentrations across various treatment groups within different experiment groups, we utilized one-way ANOVA (R software version 4.3.2) to determine the statistical significance of these disparities. A significance threshold was established at *p* < 0.05. For identifying specific differences between pairs of treatments, Tukey’s HSD post-hoc test was conducted for analysis. The median lethal concentrations (LC_50_) were calculated by the “ecotox” package of R software (version 4.3.2).

## 3. Results and Discussion

### 3.1. Cu Toxicokinetics

**Figure 1:**
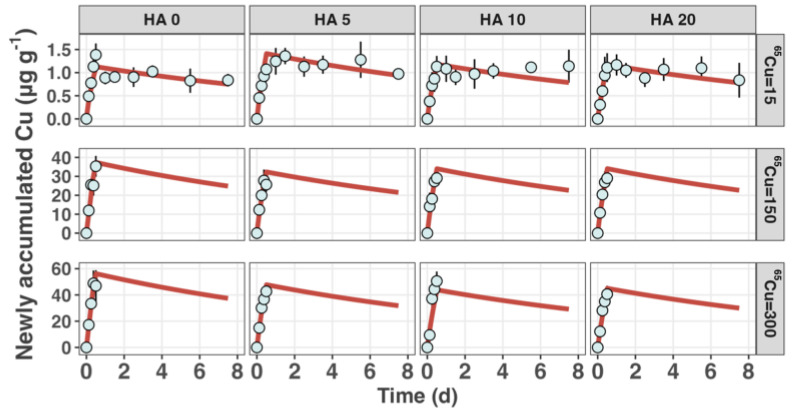
^65^Cu accumulation and elimination in the razor clam *S. constricta*. The average newly accumulated ^65^Cu (µg g^−1^) over a 12 h exposure period under varying concentrations of humic acid (HA) (nominal concentrations are 0, 5, 10, and 20 mg L^−1^) and ^65^Cu (nominal concentrations are 15, 150, and 300 µg L^−1^). The duration of the depuration period is 7 days. Error bars represent standard deviations from the mean (*n* = 6), and the depicted curves represent model fits.

The toxicokinetics of Cu in *S. constricta* were investigated using a one-compartment model, which effectively fitted the experimental data, as shown in Figure 1. During the Cu accumulation and elimination phases, the clams consistently displayed a significant increase in Cu concentration during the accumulation stage, followed by a marked decrease during the elimination stage, across the entire nominal HA concentration range from 0 to 20 mg L^−1^. Given the negligible changes in the body weight of the clams across all groups within the approximately 8-day experiment, the influence of growth on the variations in ^65^Cu concentration in clams was not included in the modeling. 

Table 1 presents the best-fit model parameters for Cu uptake and elimination, calculated at three nominal ^65^Cu concentrations (15, 150, and 300 µg L^−1^) across various HA concentrations (0, 5, 10, and 20 mg L^−1^). Pan and Wang (2009) [28] investigated Cu biokinetics in five marine bivalves using the isotope ^67^Cu, determining $k_{u}$ and $k_{e}$ values. Their results showed $k_{u}$ values ranging from 0.053 to 0.326 L g^−1^ h^−1^. In contrast, our findings at the 15 µg L^−1^ exposure level indicated higher $k_{u}$ values, ranging from 0.269 to 0.307 L g^−1^ h^−1^. Additionally, Zhong et al. (2012) [20] reported a Cu uptake rate constant of only 0.083 L g^−1^ h^−1^ for *P. viridis* at a salinity of 33, potentially indicating reduced Cu bioavailability at higher salinity [29,30].

The uptake rate constants $k_{u}$ represent values measured at nominal ^65^Cu concentrations of 15, 150, and 300 μg L^−1^, with each level corresponding to varied concentrations of humic acid (HA). A consistent value for the elimination rate constant $k_{e}$ was applied across varying levels of HA and ^65^Cu concentrations; $k_{e}$ was estimated to be 0.0582 ± 0.0139 d^−1^. Presented values are means ± standard deviation.

The elimination rate constant ( $k_{e}$ ) is viewed as independent of external environmental factors, relating solely to the organism’s biology. Thus, $k_{e}$ remains unaffected by changes in ^65^Cu concentration or HA levels in the water, leading to its consistent value in parameter calculations. The $k_{e}$ for *S. constricta* is 0.0582 ± 0.0139 d^−1^, slightly above that of the oyster *Saccostrea cucullata* (0.032 ± 0.021 d^−1^) but below that of other bivalves [28,30].

### 3.2. Cu Uptake at Different DOC Levels

Table 1 displays the variations in the uptake rate constant ( $k_{u}$ ) in response to different concentrations of HA at three ^65^Cu exposure concentrations. The actual DOC concentrations have been determined as 0.58, 3.05, 5.61, and 8.98 mg L^−1^ for each HA treatment (Table 2). In general, the $k_{u}$ s initially decreased slightly with increasing HA concentrations before stabilizing between each Cu exposure treatment. Specifically, at a DOC concentration of 0.58 mg L^−1^, the $k_{u}$ was 0.307, 0.625, and 0.481 L g^−1^ h^−1^ which slightly decreased to 0.274, 0.578, and 0.424 L g^−1^ h^−1^ at 8.98 mg L^−1^ DOC for three Cu exposure treatments, respectively. In a similar context, Sánchez-Marín et al. (2016) [31] also observed a decrease in $k_{u}$ for *Mytilus edulis* in the presence of HA, with $k_{u}$ declining from 1.939 ± 0.213 to 0.958 ± 0.068 L g^−1^ h^1^, mirroring the trend we found in our study. It is worth noting that Chen (2017) [22] reported that the $k_{u}$ of Cu for *Potamocorbula laevis* decreased from 0.0184 to 0.0156 L g^−1^ h^−1^ then increased to 0.0533 L g^−1^ h^−1^ as the HA concentration varied from 0.12 mg L^−1^ to 10.3 mg L^−1^. The author observed that precipitation occurred during the experimental processes, which may have formed complexes with Cu and been inadvertently ingested by the clams, thus being absorbed through the digestive tract. That might be the reason for $k_{u}$ increasing significantly when exposed to high HA concentrations.

**Figure 2:**
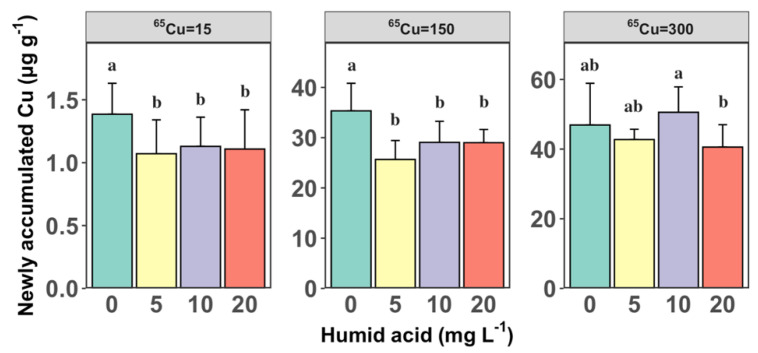
The average newly accumulated Cu (µg g^−1^) at 12 h under varying concentrations of humic acid (HA) (0, 5, 10, and 20 mg L^−1^) and three levels of ^65^Cu exposure (15, 150, and 300 µg L^−1^). Error bars represent standard deviations from the mean (*n* = 6). different letters (e.g., “a” vs. “b”) signify that there are significant differences between these groups, with a *p*-value less than 0.05.

Figure 2 depicts the 12 h Cu accumulation in *S. constricta*, highlighting the impact of HA concentration at each Cu exposure treatment. At ^65^Cu exposure levels of 15 µg L^−1^ and 150 µg L^−1^, there was a significant reduction in the ^65^Cu accumulation in clams from 1.39 ± 0.25 and 35.3 ± 5.49 µg g^−1^ to 1.07 ± 0.27 and 25.7 ± 3.77 µg g^−1^, respectively, as the actual DOC concentration significantly increased from 0.58 to 3.05 mg L^−1^ (one-way ANOVA, *p* < 0.05). This aligns with the findings of Kamunde and MacPhail (2011) [32], who also reported the reduced bioaccumulation of Cu in rainbow trout after the introduction of HA, highlighting the role of HA in lowering the bioavailability of waterborne Cu, thereby reducing its accumulation in aquatic organisms. A plausible explanation is that the addition of HA to the exposure medium significantly reduces the proportion of free Cu^2+^ ions, thereby decreasing the bioavailability of Cu.

Moreover, there was no significant change in the levels of ^65^Cu accumulation in the clams when the actual DOC concentration increased from 3.05 to 8.98 mg L^−1^ (one-way ANOVA, *p* > 0.05). This trend is similar to the variation in the $k_{u}$ s in relation to the concentration of DOC across each Cu exposure concentration. One possible interpretation of our findings is that when the actual concentration of DOC exceeds a threshold of 3.05 mg L^−1^, there is an augmented formation of colloids as a result of Cu binding with HA. While we did not observe precipitation during our experiments, as noted in a previous study [22], some studies suggest that the introduction of HA into aquatic systems can lead to colloidal metal–HA formation. These complexes could be ingested through the digestive pathways of clams, potentially leading to a rise in the overall Cu concentration within the organisms as this bound form of Cu is taken up. For example, Sánchez-Marín et al. [31] found that HA did not significantly affect Cu accumulation in the overall soft tissues of the mussel *Mytilus edulis*. In their research, like ours, the modeling method employed in their study illustrates that colloidal metal, presented as HA, can be absorbed through the gut of marine mussels. Similar conclusions were also reported in an earlier study [33]. 

The elimination rate constant ( $k_{e}$ ), like the salinity variation in the previous report [29,30], could fit well with the depuration across varying concentrations of HA, suggesting that unlike the uptake process, the elimination rate was not dependent on HA varying. The constant elimination rate implies that the observed differences in Cu accumulation are primarily driven by variations in uptake rates rather than changes in elimination dynamics.

The relationship between the uptake rate $J_{int}\left(t\right)$ and the waterborne Cu concentration $C_{w}\left(t\right)$ adheres to the Michaelis–Menten equation (Table 3). For varying actual DOC concentrations of 0.58, 3.05, 5.61, and 8.98 mg L^−1^, the maximal Cu uptake rates ( $J_{max}$ ) were calculated as 211, 202, 142, and 176 µg g^−1^ h^−1^, respectively. The half-saturation constants (*K_m_*s), indicative of the Cu concentration at which the uptake rate is half of $J_{max}$ , were determined to be 346, 230, 135, and 204 µg L^−1^.

### 3.3. Toxicodynamics and Effects of DOC

**Figure 3:**
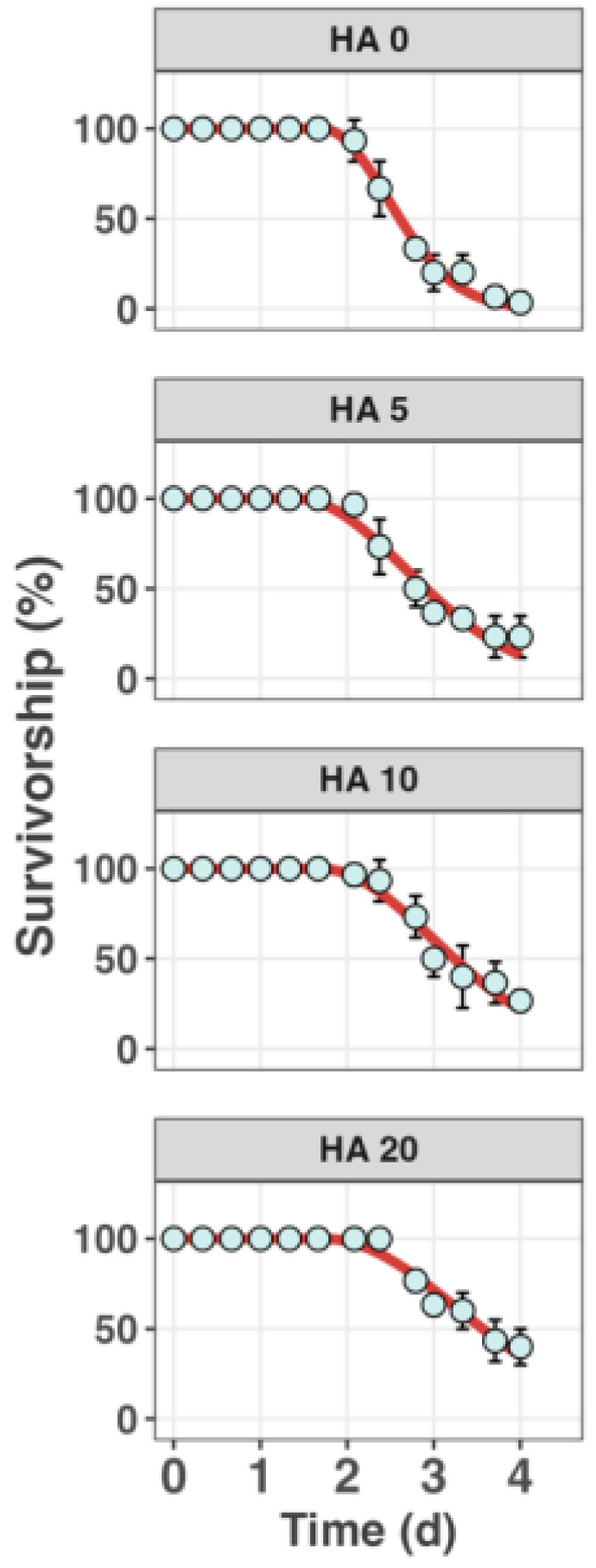
Survivorship of the razor clam *S. constricta* when exposed to 300 µg L^−1^ ^65^Cu across a gradient of humic acid (HA) (nominal concentrations are 0, 5, 10, and 20 mg L^−1^) for 96 h. Data points represent the mean observed survival percentages with error bars indicating standard deviation (*n* = 3). The lines represent the best fits from the model.

Figure 3 shows the dynamics of survivorship of *S. constricta* when exposed to 300 µg L^−1 65^Cu in conjunction with a range of DOC concentrations (0, 3.05, 5.61, and 8.98 mg L^−1^) over a period of 96 h. There was a corresponding increase in the LC_50_s of Cu from 72 µg L^−1^ to 493 µg L^−1^. Previous studies also reported adding HA in the exposure medium can effectively enhance the Cu LC_50_s in aquatic animals [13,34,35], which is consistent with our observation. Moreover, the 96 h survival rate of *S. constricta* climbed from approximately 0% to about 50% with DOC concentrations from 0.58 mg L^−1^ to 8.98 mg L^−1^. This trend also indicates that an increase in HA concentration can significantly mitigate the toxic effects of Cu on *S. constricta*.

Table 1 presents the best-fit estimates of the threshold concentration ( $C_{IT}$ ) for toxicity and the killing rate ( $k_{k}$ ) derived from the toxicodynamic (TD) model applied to the survivorship data of the clams. The $C_{IT}$ of Cu increased with the DOC concentration, rising from 104 ± 9.4 µg g^−1^ up to 140 ± 11 µg g^−1^ at 8.98 mg L^−1^ DOC. The killing rate, $k_{k}$ , showed a decreasing trend from 10.4 ± 1.89 mg µg^−1^ h^−1^ to 5.68 ± 1.08 mg µg^−1^ h^−1^ with increased DOC concentrations, indicating that the grams of *S. constricta* killed by per mg of internalized Cu per hour were reduced. The calculated variations in these parameters potentially reflect a protective effect of HA against Cu toxicity. Our results are similar to the $C_{IT}$ determined in a previous study which was 92 g µg^−1^ in *P. laevis* at a salinity of 15, and the $k_{k}$ s were lower than ours which was 21.8 mg µg^−1^ h^−1^ [30]. Under the three Cu exposure concentrations, when the actual concentration of HA exceeded 3.5 mg L^−1^, there were no significant changes in the accumulated amount of Cu over 12 h. This suggests that the threshold concentration did not increase in a regular pattern with rising HA levels, and the killing rate decreased as the HA concentration increased. This may indicate that the toxicity of Cu–DOC complexes absorbed through the digestive tract is lower than that of Cu absorbed directly from the water.

## 4. Conclusions

In conclusion, our study demonstrated that the impact of HA on the bioaccumulation and toxicity of Cu for *S. constricta* manifests in two main ways: it alters the distribution of Cu species and affects the uptake pathways of Cu. When the actual DOC concentration is below a certain threshold, it primarily reduces Cu bioavailability by altering its speciation, thus diminishing Cu bioaccumulation. However, at higher DOC concentrations, HA may promote the formation of colloidal Cu–HA complexes in the solution, which could then be ingested by *S. constricta*. Moreover, beyond the commonly acknowledged mechanism of diminishing Cu accumulation and toxicity for bivalves through the formation of Cu–DOC complexes, we propose that the Cu–DOC complexes ingested via the digestive tract might be less toxic than Cu absorbed directly from the water. This study highlights the importance of considering both chemical and biological factors in evaluating the health of marine ecosystems and the safety of seafood.

## Figures and Tables

**Table 1:** Best-fit values of the model parameters.

Humic Acid (mg L^−1^)	$k_{u}$ (15) (L g^−1^ h^−1^)	$k_{u}$ (150) (L g^−1^ h^−1^)	$k_{u}$ (300) (L g^−1^ h^−1^)	$C_{IT}$ (µg^−1^ g^−1^)	$k_{k}$ (mg µg^−1^ h^−1^)
0	0.307 ± 0.020	0.625 ± 0.044	0.481 ± 0.033	104 ± 9.40	10.6 ± 1.89
5	0.276 ± 0.011	0.611 ± 0.032	0.469 ± 0.024	117 ± 11.7	8.31 ± 1.73
10	0.269 ± 0.019	0.599 ± 0.058	0.406 ± 0.040	130 ± 10.1	8.32 ± 1.65
20	0.274 ± 0.009	0.578 ± 0.027	0.424 ± 0.020	140 ± 11.0	5.68 ± 1.08

The uptake rate constants *k_u_* represent values measured at nominal ^65^Cu concentrations of 15, 150, and 300 μg L^−1^, with each level corresponding to varied concentrations of humic acid (HA). A consistent value for the elimination rate constant *k_e_* was applied across varying levels of HA and ^65^Cu concentrations; *k_e_* was estimated to be 0.0582 ± 0.0139 d^−1^. Presented values are means ± standard deviation.

**Table 2:** Measured concentrations of DOC (mg L^−1^) in the exposure seawater of different HA levels used in the experiments.

Nominal (mg L^−1^)	0	5	10	20
Measured (mg L^−1^)	0.58	3.05	5.61	8.98

**Table 3:** The 96 h median lethal concentration (LC_50_) of Cu in the clam *S. constricta* determined at four different humic acid (HA) levels (0, 5, 10, and 20 mg L^−1^). The relationship between uptake rates $J_{int}$ of Cu and Cu concentrations in exposure solution ([Cu], ug L^−1^) are described by the Michaelis–Menten equation.

Humic Acid (mg L^−1^)	96 h LC_50_ (µg L^−1^)	$J_{int}$ (µg g^−1^ h^−1^)
0	72	211 × [Cu]/(346 + [Cu])
5	128	202 × [Cu]/(230 + [Cu])
10	276	142 × [Cu]/(135 + [Cu])
20	493	176 × [Cu]/(204 + [Cu])

## Data Availability

Data are contained within the article.

## Supplementary Materials

The following supporting information can be downloaded at: www.mdpi.com/xxx/s1, Table S1: Measured concentrations of ^65^Cu (μg L^−1^) in the exposure seawater of different humic acid used in the accumulation experiment.
