# Supplementary material for: Investigating the Impact of Humic Acid on Copper Accumulation in Sinonovacula constricta Using a Toxicokinetic–Toxicodynamic Model
Source: Toxics. 2024 Jan 15;12(1):0. doi: 10.3390/toxics12010074 (PMC11154395; doi:10.3390/toxics12010074)
Supplement: Supplementary file 1 [file toxics-12-00074-s001.zip › toxics-2810666-supplementary.pdf]

Supporting Information

Table S1. Measured concentrations of ^{65}Cu (μg L^{-1}) in the exposure seawater of different humic acid used in the accumulation experiment.

Nominal ^{65}Cu concentration = 15 μg L^{-1}				
Time (h)	Humic acid (mg L^{-1})			
	0	5	10	20
0	13.3	11.8	11.7	11.2
3	8.6	10.0	9.1	8.9
6	7.4	8.7	7.9	8.9
9	6.9	8.4	7.5	7.2
12	5.9	7.3	6.5	6.7

Nominal ^{65}Cu concentration = 150 μg L^{-1}				
Time (h)	Humic acid (mg L^{-1})			
	0	5	10	20
0	136	146	143	134
3	125	113	128	123
6	148	109	110	110
9	94	86	97	107
12	84	80	91	104

Nominal ^{65}Cu concentration = 300 μg L^{-1}				
Time (h)	Humic acid (mg L^{-1})			
	0	5	10	20
0	268	257	241	260
3	224	216	203	223
6	201	197	182	202
9	197	169	161	186
12	178	141	149	171
